# Poland Syndrome and Multimorbidity in a 79-Year-Old Male

**DOI:** 10.7759/cureus.63349

**Published:** 2024-06-28

**Authors:** Ning Zhang, Xuan Qu, Lin Kang, Xiaohong Liu

**Affiliations:** 1 Department of Geriatrics, Peking Union Medical College Hospital, Peking Union Medical College, Chinese Academy of Medical Sciences, Beijing, CHN

**Keywords:** poland syndrome, older adult, comprehensive geriatric assessment, multimorbidity, chronic pain management

## Abstract

This case study highlights a 79-year-old man with chronic low back pain attributed to severe lumbar scoliosis. Physical examination revealed the unilateral absence of pectoral muscles and ipsilateral hand anomalies, indicative of Poland syndrome (PS). The patient also experienced depression due to chronic pain and PS-related anomalies. A multi-disciplinary approach proved effective in alleviating both pain and depression.

## Introduction

Poland syndrome (OMIM 173800), first described in 1841 by Alfred Poland, is characterized by the unilateral absence (partial or complete) of the pectoralis major muscle and ipsilateral syndactyly and brachydactyly. Associated abnormalities include neurologic, otologic, genitourinary, gastrointestinal, cardiovascular, and hematologic anomalies, such as congenital bilateral facial palsy, deformities of the external ear, renal agenesis, hypoplasia and ureteral reflux, thoracic cage defects, diaphragmatic hernia, herniation of lung, scoliosis, dextrocardia, axillary web or band, spherocytosis, and malignancy [1]. The exact etiology of Poland syndrome (PS) remains unknown. However, the prevailing theory suggests an interruption of the embryonic blood supply of the upper limb bud due to hypoplasia of the ipsilateral subclavian artery or its branches [2]. Here we present a rare case of chronic low back pain in a 79-year-old man with PS, lumbar scoliosis, and diaphragmatic hernia, who had not been previously diagnosed with PS. Furthermore, multimorbidity presents a significant challenge in this case. To our knowledge, this is possibly the oldest patient diagnosed with PS.

## Case presentation

A 79-year-old man presented in August 2023 with a three-year history of low back pain, accompanied by lower extremity muscular fatigue and an unstable gait. The patient described the pain as occasionally unbearable and also reported experiencing depression and anxiety. His past medical history revealed a medial meniscectomy of the left knee in 1966 following a trauma, with no significant familial history.

**Figure 1:**
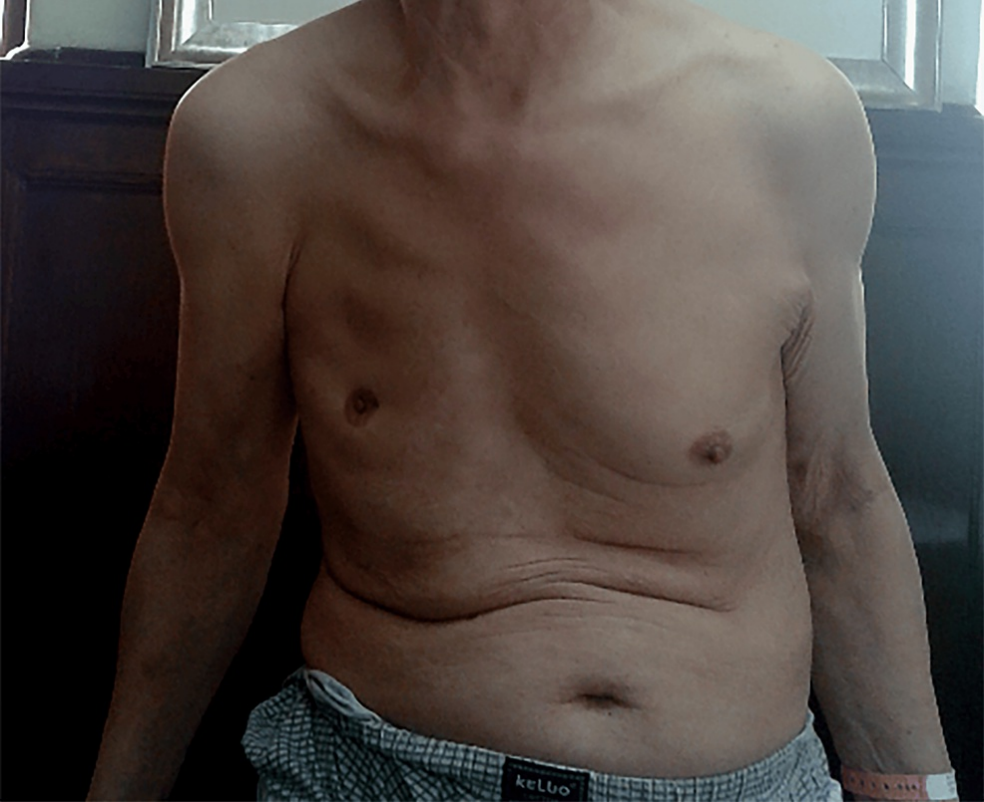
Features corresponding to Poland syndrome in our patient. Depression of the right anterior chest wall, with the absence of the right pectoralis major muscle.

**Figure 2:**
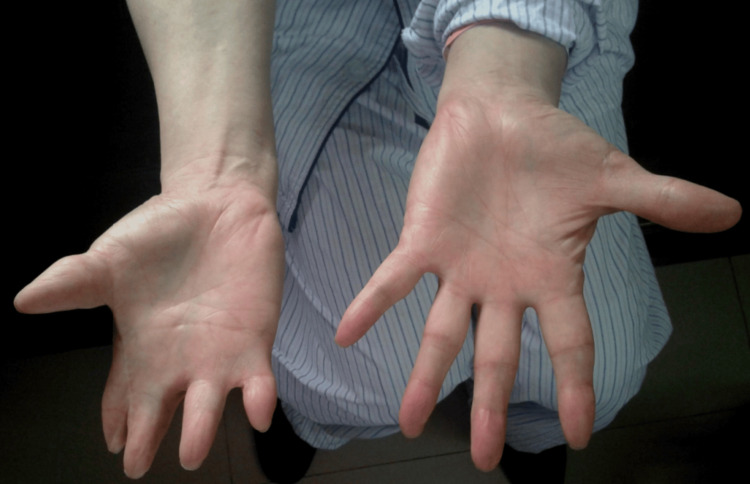
Hypoplastic right hand with syndactyly and brachydactyly of the right hand, with a normal left hand (palms of hands).

**Figure 3:**
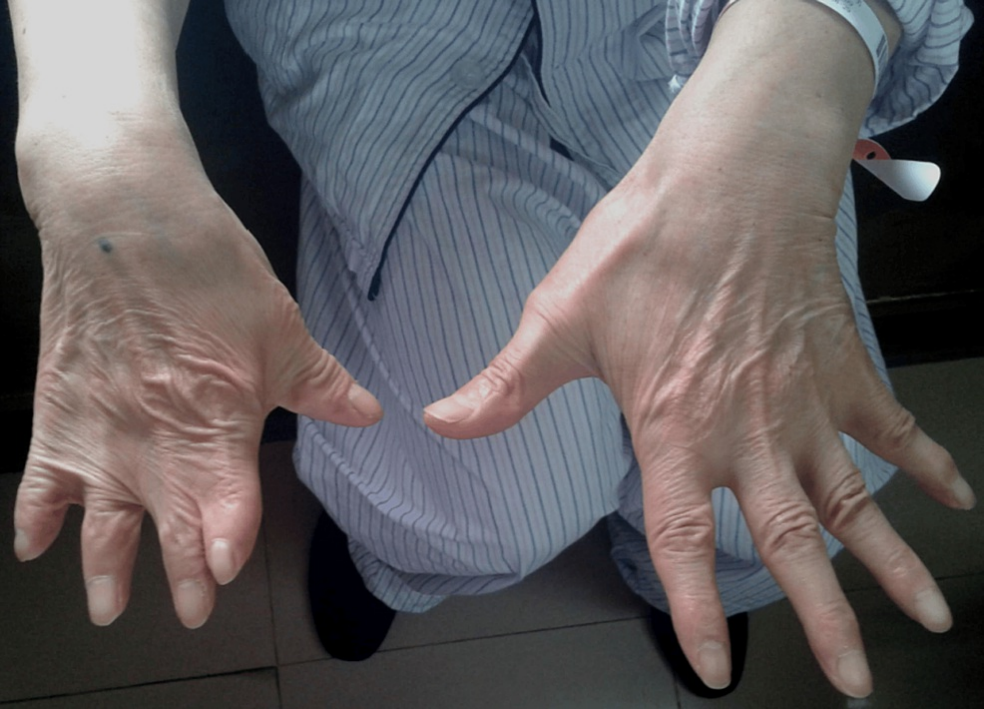
Hypoplastic right hand with syndactyly and brachydactyly of the right hand, with a normal left hand (back of hands).

Physical examination revealed depression of the right anterior chest wall, along with the absence of the right pectoralis major muscle (Figure 1). Additionally, the right hand displayed hypoplasia, brachydactyly in all fingers, and syndactyly involving the index, middle, ring, and little fingers (Figure 2 and Figure 3). The patient also presented with significant lumbar scoliosis. Cardiorespiratory and abdominal examinations did not show any abnormalities. The patient reported that he never developed pectoral muscles on the right side and had noticed anomalies in his right hand since birth. Despite this, he had not sought medical attention for these issues. The patient had been aware of his scoliosis for over a decade, but it was only in the last three years that he began experiencing low back pain.

**Figure 4:**
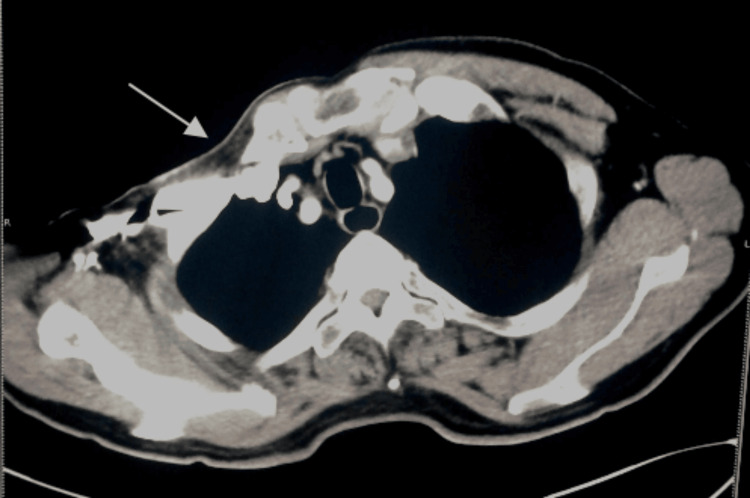
Axial plain CT scan shows pectoral muscles on the left chest but pectoral muscles are absent on the right side (arrows).

**Figure 5:**
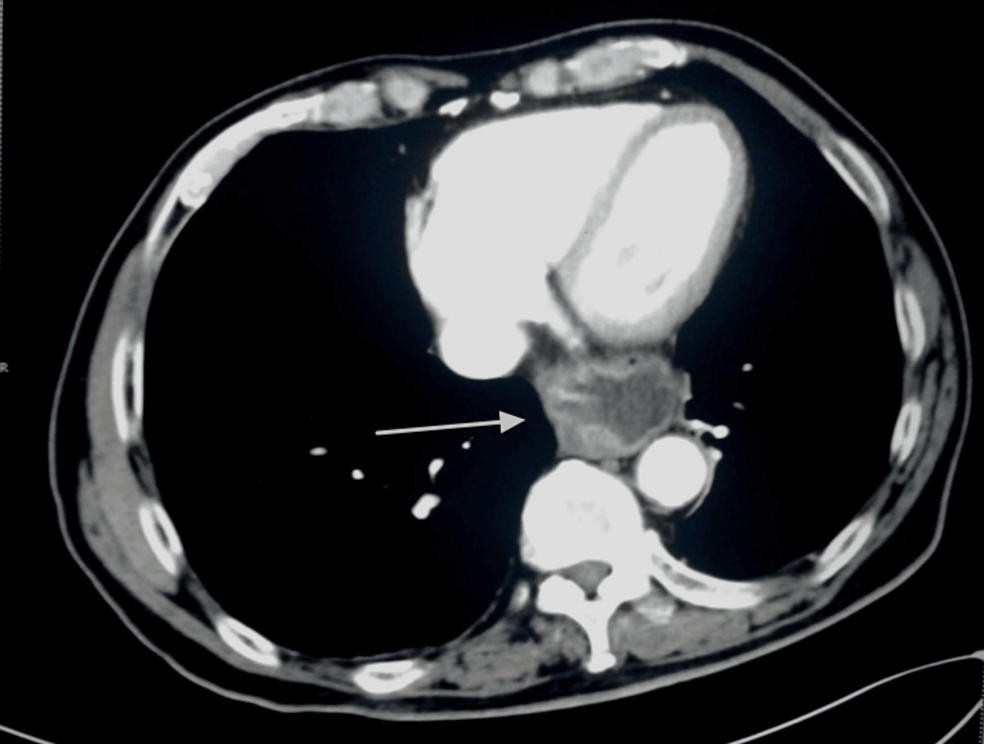
Axial plain CT scan shows diaphragmatic hernia (arrows).

**Figure 6:**
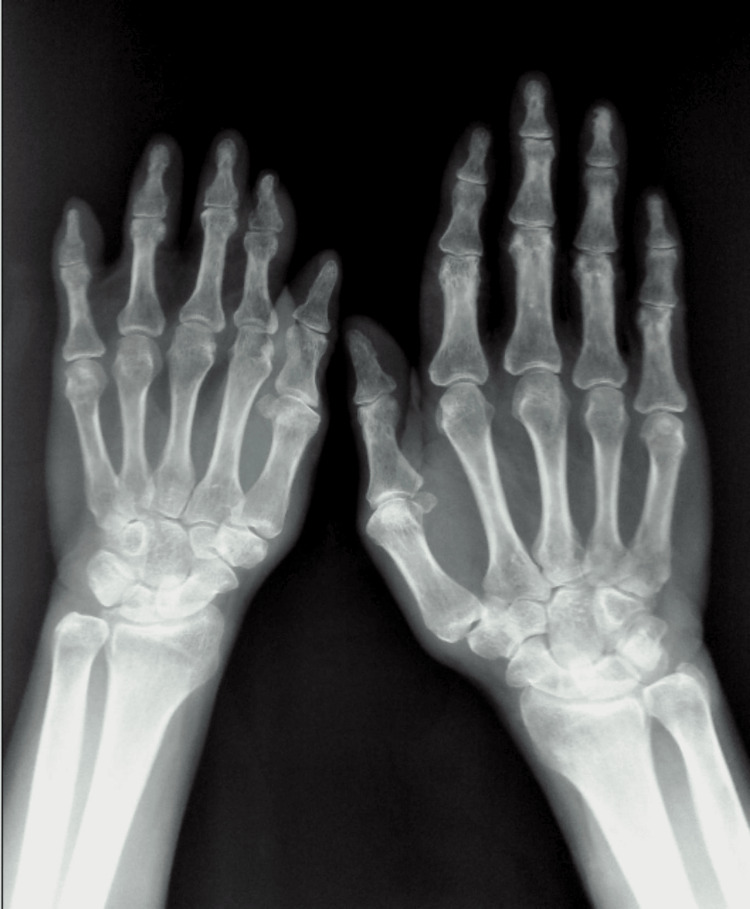
X-ray of hands showing hypoplasia of middle and distal phalanges of index, ring, middle, and little fingers of the right hand, with a normal left hand.

**Figure 7:**
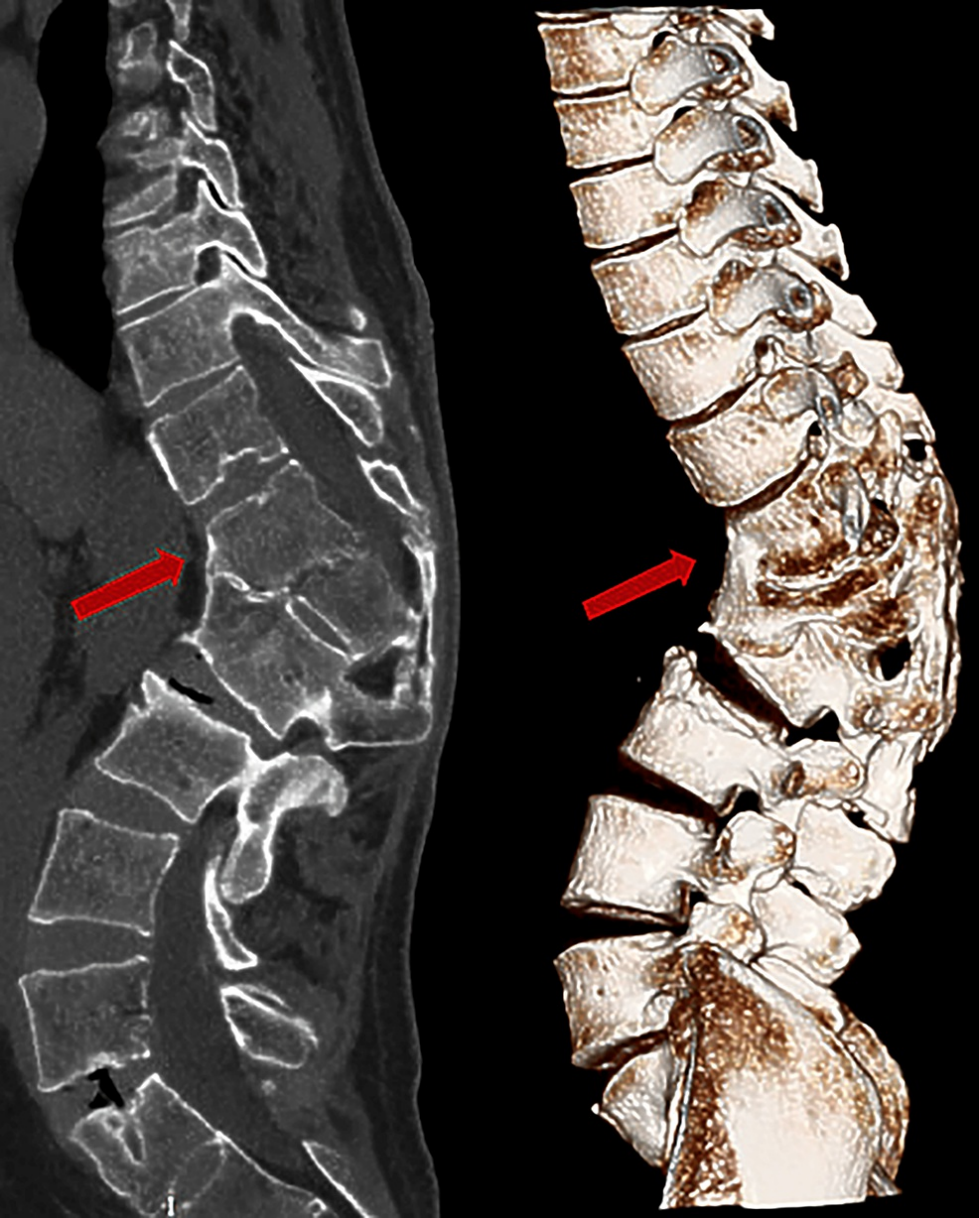
The patient's spine MRI (left) and spine CT three-dimensional reconstruction (right) showed severe scoliosis (arrows).

Laboratory findings were within normal limits. A thoracic computed tomography (CT) scan identified the absence of the right pectoralis major and minor muscles (Figure 4) along with the presence of a diaphragmatic hernia (Figure 5). X-ray examination of the right hand showed hypoplasia of the middle and distal phalanges of the index, middle, ring, and little fingers (Figure 6). Chest X-ray did not reveal any rib anomalies. Lumbar magnetic resonance imaging (MRI) revealed lumbar scoliosis with bulging of degenerative intervertebral discs in the L2-S1 region and thickening of the ligamentum flavum. Additionally, spine CT three-dimensional reconstruction confirmed the presence of severe lumbar scoliosis (Figure 7).

The diagnosis of PS was confirmed based on the presence of ipsilateral absence of pectoral muscles and hand anomalies. Following admission, a Comprehensive Geriatric Assessment (CGA) was performed, indicating a Mini-Mental State Examination (MMSE) score of 29 out of 30, an activity of daily living scale (ADL) score of 5 out of 6, and an instrumental activity of daily living Scale (IADL) score of 2 out of 8. The CGA indicated a decrease in the patient's grip strength and walking speed (left hand grip strength 19kg, walking speed 0.5m/s), as well as reduced lower limb muscle strength, poor balance ability, and a high risk of falling. The patient experienced sleep disturbances due to pain and expressed feelings of inferiority dating back to adolescence. A score of 8 on the 15-item Geriatric Depression Scale (GDS 15) indicated depression. The Geriatric Interdisciplinary Team (GIT), consisting of a geriatrician, a rehabilitation therapist, a psychologist, and a clinical pharmacist, collaborated to create intervention plans for the patient. The patient initially received tramadol 100 mg/day orally, which was later increased to 100 mg every 12 hours after five days. This adjustment led to a significant reduction in the patient's back pain, with the numeric rating scale (NRS) score decreasing from 5-7 points upon admission to 2-3 points. Regarding the patient's mood, the psychologist diagnosed depression and prescribed 60 mg of duloxetine enteric-coated capsules once daily orally. The patient exhibits decreased muscle strength in the lower limbs, poor balance function, and a high risk of falling. The rehabilitation therapist designed a training program focusing on improving lower limb muscle strength and enhancing balance. Lower limb muscle strength training includes quadriceps isometric contraction exercises (straightening the legs, tightening the thigh muscles for five seconds, relaxing, repeating 10 times to strengthen the muscles around the knee) and sit-and-leg-raise exercises (sitting upright, withdrawing the left foot for support, slowly raising the right leg, holding for three seconds, repeating 10 times to work on hip and thigh muscles). Lower support balance positioning training involves standing with feet shoulder-width apart, arms hanging naturally, and knees and hips slightly bent. Weight transfer exercises from left to right and front and back weight transfer exercises with hands gently wrapped around the abdomen were performed for 30 seconds, three times per set, and three sets per day. Surgical correction for scoliosis and associated anomalies related to PS was considered inappropriate due to the patient's poor overall health. Upon discharge from the hospital, the GIT team advised the patient to make aging-friendly adjustments at home, such as installing anti-slip handrails near the toilet and shower, as well as placing anti-slip mats in the bathroom.

Following a three-week hospital stay, the patient had a follow-up visit one month after discharge. During this visit, the patient had a significant improvement in mood and sleep quality. The retest Geriatric Depression Scale (GDS) score was 3, and the pain score on the NRS ranged from 1 to 2. Additionally, the patient's family members followed the GIT team's recommendations and made anti-fall and aging-friendly modifications in the elderly individual's living space.

## Discussion

The incidence of PS has been estimated to occur between 1 in 30,000 and 1 in 80,000 live births [3]. It is more common in males and typically affects the right side of the body [4]. Most cases are sporadic, with only a few being familial. The inheritance pattern may be autosomal dominant, with varying degrees of symptoms and reduced likelihood of manifestation. Our case highlights that PS may go undiagnosed, either due to lack of obvious disability or limited access to healthcare resulting from poverty and low socioeconomic status. This syndrome can also be concurrent with conditions like scoliosis or diaphragmatic hernia. Scoliosis in PS is often attributed to muscular imbalances in the shoulder girdle [5]. Additionally, the co-occurrence of congenital diaphragmatic hernia and PS is thought to be more than coincidental, possibly sharing a common developmental origin or laterality defect [6]. In our case, both scoliosis and diaphragmatic hernia were present, although it remains unclear if this association is purely coincidental. Severe finger and chest wall abnormalities in PS can lead to not only cosmetic concerns but also emotional challenges such as depression and feelings of inferiority, as observed in our case. Reconstructive surgeries are typically necessary for aesthetic reasons, but it is equally important to address any emotional distress stemming from the associated anomalies, providing appropriate treatment when needed. Early intervention for the deformities associated with PS is crucial to prevent long-term complications as they become apparent.

Multimorbidity, encompassing congenital anomalies, chronic pain, depression, and somatoform disorders, is the central focus of this study. Major consequences of multimorbidity include functional impairment, reduced quality of life, and increased healthcare utilization and costs [7]. Therefore, it is essential to conduct a CGA to identify and document these issues. The CGA is a comprehensive interdisciplinary diagnostic process aimed at assessing the medical, psychological, and functional abilities of frail elderly individuals to develop a well-coordinated treatment plan and long-term monitoring [8]. It is important to highlight that chronic low back pain resulting from degenerative lumbar scoliosis is a significant debilitating issue in this context. Unfortunately, it is often overlooked and underestimated as a health concern in the elderly population. The adverse effects of pain in the elderly are manifold, with depression being a common occurrence among elders experiencing pain [9]. Furthermore, social isolation, sleep disturbances, decreased mobility, reduced enjoyment of life, and changes in social interactions are additional challenges faced by elders living with pain [10]. The primary objective in treating degenerative lumbar scoliosis is to alleviate pain and enhance functional abilities with minimal intervention [11]. In this particular case, a multidisciplinary approach was adopted, involving pain and depression management, as well as physical rehabilitation, resulting in positive outcomes. Surgical intervention was deemed unsuitable due to the patient's poor overall health condition. It is crucial to emphasize that maintaining functional capacity should be a key goal in the care of geriatric patients with multimorbidity.

## Conclusions

In conclusion, PS is a rare congenital disorder that should be considered. It is a condition present from birth with various manifestations. It is important to conduct a thorough examination as the disorder can be easily missed. Utilizing a CGA is beneficial in managing medication for older individuals. It is crucial to address emotional disturbances, particularly depression, in elderly patients with chronic illnesses. Providing multidisciplinary care, as seen in our patient's case, can help alleviate pain, enhance mood, strengthen muscles in the lumbar and lower limbs, and ultimately improve overall function. It is imperative to remember that medical care for the elderly should prioritize the individual over the disease.
